# Early Sepsis Diagnosis as a Global Imperative: The Role of Raman Spectroscopy

**DOI:** 10.3390/jcm15083138

**Published:** 2026-04-20

**Authors:** Andrea Piccioni, Fabio Spagnuolo, Marina Sebastiani, Alberto Valentini, Giuseppe Pezzotti, Marcello Candelli, Marcello Covino, Marco De Spirito, Antonio Gasbarrini, Francesco Franceschi

**Affiliations:** 1 Department of Emergency Medicine, Università Cattolica del Sacro Cuore, 00168 Rome, Italy; fabio.spagnuolo01@icatt.it (F.S.); marina.sebastiani01@icatt.it (M.S.); marcello.candelli@policlinicogemelli.it (M.C.); marcello.covino@policlinicogemelli.it (M.C.); marco.despirito@unicatt.it (M.D.S.); antonio.gasbarrini@policlinicogemelli.it (A.G.); 2 Department of Emergency Medicine, Fondazione Policlinico Universitario Agostino Gemelli-IRCCS, 00168 Rome, Italy; alberto.valentini@policlinicogemelli.it; 3 Biomedical Engineering Center, Kansai Medical University, 1-9-11 Shin-machi, Hirakata 573-1191, Osaka, Japan; pezzotti.giu@kmu.ac.jp; 4 Medical and Surgical Science Department, Fondazione Policlinico Universitario Agostino Gemelli-IRCCS, 00168 Rome, Italy

**Keywords:** sepsis, diagnosis, Raman spectroscopy, infection, immunity, pathogens, blood culture

## Abstract

**Background/Objectives:** Sepsis is a leading cause of hospital mortality and represents a time-sensitive medical emergency. Current diagnostic strategies rely on clinical assessment, severity scores, biomarkers, and blood cultures. However, blood cultures require 24–72 h for pathogen identification and demonstrate limited sensitivity, while biomarkers such as procalcitonin and C-reactive protein lack optimal specificity. These limitations support the widespread empirical use of broad-spectrum antibiotics and highlight the need for rapid, sensitive, and culture-independent diagnostic tools. **Methods:** A narrative literature review was conducted using PubMed and Google Scholar, including 28 studies published over the past 10 years, encompassing observational and preclinical investigations. Current evidence on the application of Raman spectroscopy in sepsis was summarized, with a dual focus on pathogen identification and the assessment of the host response. **Results:** Raman spectroscopy has demonstrated the ability to detect early molecular alterations in circulating immune cells and mitochondrial redox status, potentially preceding conventional biomarkers. For pathogen identification, Raman techniques have achieved diagnostic accuracies comparable to automated systems, but with significantly shorter turnaround times. Integration with microfluidics, optical tweezers, and deep learning algorithms has further enhanced performance, although these applications remain largely experimental. **Conclusions:** Despite these promising results, the lack of methodological standardization, spectral overlap among phylogenetically related species, limited large-scale validation, and challenges in interpreting certain spectral signatures remain unresolved. Most available evidence originates from preclinical, single-center, and controlled studies, underscoring the need for prospective multicenter trials and harmonized protocols.

## 1. Introduction

Sepsis remains one of the leading causes of mortality among hospitalized patients and constitutes a true time-dependent medical emergency. Its global epidemiological burden is still substantial; in 2021, at least 166 million cases and 21.4 million sepsis-related deaths were reported, accounting for approximately 31.5% of all deaths worldwide (with a disproportionate impact in low-income countries) [1]. Bacterial infections continue to predominate over fungal and viral etiologies.

Sepsis is currently defined as a life-threatening organ dysfunction caused by a dysregulated host response to infection [2]. Despite advances in intensive care medicine, early diagnosis remains a major clinical challenge, particularly in the initial stages of disease, when signs and symptoms may be nonspecific or atypical. Fever, hypothermia, altered mental status, or hemodynamic instability may be mild or subtle, especially in elderly, immunocompromised, or multimorbid patients, making timely recognition difficult. Septic patients may also present with normothermia, elevated troponin levels, hypotension, syncope, confusion, or psychomotor agitation [2,3,4]. Thus, clinicians continue to face a significant diagnostic challenge, still insufficiently supported by tools capable of reliably enabling early detection. Sepsis is a time-dependent syndrome. Observational studies and international guidelines consistently emphasize that early recognition and prompt administration of appropriate antibiotics are associated with reduced morbidity and mortality [5]. In this context, early diagnosis is not merely prognostic but plays a pivotal role in leading therapeutic decision-making.

Current diagnostic approaches rely on the integration of clinical assessment, validated scoring systems, and laboratory biomarkers, with the aim of identifying infection-related organ dysfunction at an early stage. The principal reference system is the SOFA (Sequential Organ Failure Assessment) score, which quantifies dysfunction across six organ systems (respiratory, cardiovascular, hepatic, coagulation, renal, and neurological). Sepsis is defined as a suspected or documented infection associated with an acute increase of at least two points in the SOFA score [4]. In the emergency department, the qSOFA (quick SOFA) is frequently used as a rapid bedside tool, evaluating three simple parameters: altered mental status, respiratory rate ≥22 breaths/min, and systolic blood pressure ≤100 mmHg [6]. The presence of at least two criteria identifies patients at increased risk of adverse outcomes and needs further evaluation. Other scoring systems, such as APACHE (Acute Physiology and Chronic Health Evaluation) and the NEWS (National Early Warning Score), were developed primarily as predictors of mortality and need for intensive care admission [7]. A NEWS ≥5 in a patient with suspected infection is considered highly suggestive of an increased risk of sepsis or impending septic shock [8].

Together with clinical evaluation, laboratory biomarkers play an important supportive role in diagnosis and monitoring. More than 200 biomarkers have been investigated to date; however, a true “holy grail” combining optimal sensitivity and specificity has yet to be identified. Among the most used markers, procalcitonin (PCT) demonstrates sensitivity and specificity of approximately 80%, and it is generally considered more accurate than C-reactive protein (CRP) for the diagnosis of sepsis [9]. Both the Society of Critical Care Medicine and the Infectious Diseases Society of America recognize the value of PCT, while recommending that it should not be used as the sole parameter for initiating antibiotic therapy [5]. In contrast, CRP has lower specificity and a higher false-positive rate, limiting its diagnostic utility. The combined assessment of presepsin (PSP) and PCT has been shown to improve predictive accuracy for bacteremia [10]. Finally, monocyte distribution width (MDW) appears particularly useful as an early screening parameter in the emergency department, thanks to its high negative predictive value [11].

**Figure 1:**
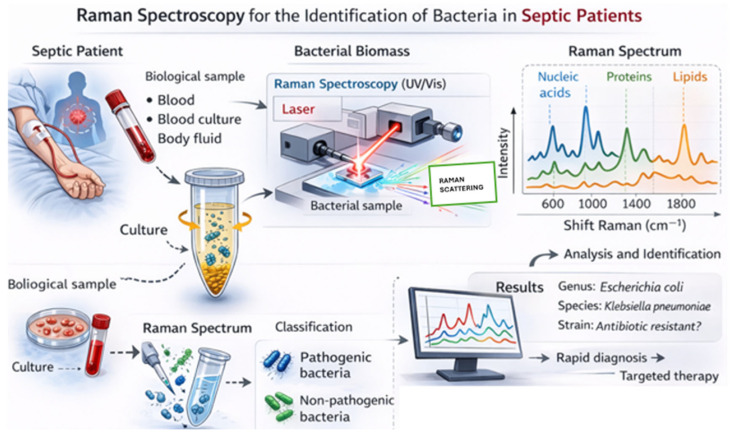
Raman Spectroscopy.

The current clinical approach frequently relies on the empirical initiation of broad-spectrum antibiotic therapy while awaiting identification of the causative pathogen. However, in the era of precision medicine, there is a growing need to move beyond this paradigm in favor of early, targeted antimicrobial therapy, with the dual aim of improving clinical outcomes and limiting the emergence of antimicrobial resistance. Blood cultures remain the gold standard for pathogen identification in sepsis. Despite a high specificity, estimated at around 95%, microbial growth and identification typically require 24–72 h, a timeframe often incompatible with the urgency of clinical decision-making [12,13]. Moreover, their sensitivity is variable and may be affected by low bacterial load, insufficient blood volume, prior antibiotic administration, or the presence of fastidious organisms. Several studies have shown that only approximately one-third of septic patients have positive blood cultures despite clear evidence of active infection, underscoring the limited sensitivity of this technique [5]. These limitations highlight a persistent unmet clinical need: rapid diagnostic tools capable of identifying pathogens directly at the bedside, with high sensitivity and specificity, ideally during the earliest phases of emergency department screening. In this context, Raman spectroscopy has emerged as a promising technology for the rapid etiological diagnosis of sepsis (Figure 1).

ChatGPT-5.3 (OpenAI) was prompted to generate this image based on our specifications, with the aim of illustrating the Raman process in an ideal point-of-care setting. The image highlights the technique’s broad applicability across all biological fluids, as well as its sensitivity to pathogens and diverse cellular populations. The Raman bands are displayed on the computer monitor.

## 2. Materials and Methods

This narrative review aims to summarize the current evidence on Raman spectroscopy, with a specific focus on its diagnostic application in sepsis. Particularly, the objective is to promote knowledge and encourage the standardization of Raman spectroscopy for the identification of causative pathogens in sepsis. A comprehensive literature search was conducted in PubMed and Google Scholar using the following keywords: “sepsis and Raman spectroscopy”, “Raman spectroscopy and Gram-positive”, “Raman spectroscopy and Gram-negative”, and “Raman spectroscopy and bacteremia”. The reference lists of retrieved articles were also screened to identify additional eligible studies. We included studies published in the past 10 years, excluding non-English publications, pediatric populations, and studies not focused on the evaluation of immune cells or blood pathogens. Approximately 120 articles were initially screened, and 28 studies were ultimately included.

To obtain the 28 studies included in the Results (Section 4), narrative and systematic reviews, meta-analyses, and articles for which only the abstract was accessible were excluded. Additional macro-categories were also excluded, as detailed below. These studies reported elements that were either overly specific, highly technical or engineering-focused, or not sufficiently aligned with the specific topic of non-viral infections and sepsis, investigated using Raman spectroscopy. 

The study selection process was guided by a specific focus on the application of Raman spectroscopy to the diagnosis and characterization of non-viral infections and sepsis, with particular emphasis on clinically relevant and potentially translatable approaches. Within this framework, a substantial number of studies were excluded due to limited alignment with the defined scope.

Firstly, studies addressing infectious conditions outside the targeted domain were excluded, including those focused on viral infections such as *SARS-CoV-2* or on parasitic diseases such as *malaria*. In addition, investigations centered on highly specific clinical contexts (such as sepsis in pregnancy or on organ-specific complications like liver cirrhosis, sepsis-associated lung or hepatic injury) were not considered, as they did not provide generalizable insights into systemic, non-viral infectious processes from a diagnostic perspective.

A second major group of exclusions comprised studies in which Raman or other spectroscopic techniques were applied to non-infectious conditions or to biological questions only indirectly related to infection or sepsis. These included analyses of hepatic vitamin A and retinoid content, hemoglobin-related parameters, capillary refill dynamics, cardiomyopathy monitoring, multiple myeloma characterization, and bone-related alterations. Despite their methodological relevance, such studies did not contribute directly to the identification or clinical management of infectious diseases.

Furthermore, other investigations were excluded due to their predominantly fundamental or mechanistic focus. These studies explored biochemical and molecular processes, such as heme–protein interactions, spectroscopic quantification of heme and its degradation products, enzymatic inhibition, or lipopolysaccharide-induced changes in monocytic cell lines, without establishing a clear link to diagnostic applications in infection or sepsis. While scientifically valuable, these contributions remain distant from the translational objectives of the present work.

Additional exclusions involved studies primarily oriented toward technological development or material science, including the design and characterization of nanoparticle-based drug delivery systems, polyplex micelles, or novel bioactive compounds. In these cases, Raman spectroscopy was often employed as an analytical tool rather than as a diagnostic modality, thereby falling outside the intended clinical scope.

Finally, studies focusing on microbiological, ecological, or methodological aspects without direct diagnostic applicability were also excluded. These included investigations of bacterial communities in non-human models, morphological characterization of biofilms, antibiotic susceptibility testing on cultured isolates, and spectroscopic differentiation of leukocyte subtypes. Similarly, works addressing antibiotic quantification in blood or monitoring of therapeutic interventions did not meet the inclusion criteria, as they did not directly target the detection or characterization of infection or sepsis.

The evidence was synthesized to provide an overview of the principles of Raman spectroscopy, its application to pathogens and immune cells, current fields of application, its potential role in sepsis, as well as its limitations, advantages and future perspectives. The distribution of the studies included in the review is shown in Table 1. This review adopts a clinically oriented perspective. Technical aspects are presented to facilitate comprehension, while readers seeking in-depth mathematical and physical details are referred to the cited references. ChatGPT-5.3 (OpenAI) was used for the generation of Figure 1 and the graphical abstract.

## 3. Raman Spectroscopy

Raman spectroscopy is a nondestructive analytical technique based on laser-matter interactions, able to provide a true “molecular fingerprint” of biological samples. The excitation source is typically a monochromatic laser operating in the visible or near-infrared range (commonly 532 nm, 785 nm, or 1064 nm) [14]. Thanks to its expertise in identifying the biochemical composition of cells and microorganisms without the need for labels or dyes, Raman spectroscopy offers direct and rapid, label-free analysis. It is classified as a vibrational spectroscopic technique, as it provides information on molecular vibrations, rotations, and other bond-related properties by detecting frequency shifts generated when photons interact with matter.

In 1928, Chandrasekhara Venkata Raman first experimentally observed the inelastic scattering of light, a phenomenon that now bears his name [15]. Spontaneous Raman scattering is an inelastic scattering process in which the interaction between an incident photon and a molecular or crystalline system results in a net exchange of energy [16]. Unlike elastic (Rayleigh) scattering, the scattered photon exhibits an energy different from that of the incident photon, reflecting transitions between internal energy states of the system. This inelastic light scattering (known as the Raman effect) enables the extraction of a molecule-specific spectral fingerprint.

In isolated molecules, the process involves transitions between roto-vibrational states. Interaction with the electromagnetic field may alter the vibrational and/or rotational state of the molecule, resulting in a frequency shift in the scattered radiation. In crystalline solids, energy exchange involves collective lattice excitations. The quantum of lattice vibration is described as a phonon, the quasiparticle associated with quantized lattice oscillations [17]. In such systems, Raman scattering may also involve other collective excitations, including surface plasmons, spin waves, or excitations associated with paramagnetic centers [18].

Depending on the energy balance between photon and material system, two principal contributions are distinguished. In the Stokes process, the scattered photon has lower energy than the incident photon, as part of the energy is transferred to the system, promoting a vibrational or lattice excitation. In the anti-Stokes process, the scattered photon has higher energy, since the system transfers energy to the electromagnetic field, typically from a thermally populated vibrational state [19].

At the microscopic level, the Raman effect arises from the dependence of the system’s electrical response on vibrational coordinates. Under equilibrium conditions, the electronic distribution is stationary; however, in the presence of an oscillating external electric field, it can be perturbed. In molecules, this response is quantified by polarizability, whereas in extended media it is described by dielectric susceptibility. Within the classical framework, Raman scattering occurs when molecular or lattice vibrations modulate the system’s polarizability (or dielectric susceptibility) over time [20]. This modulation introduces additional frequency components into the scattered field, corresponding to the experimentally observed Raman shifts.

The vibrational frequencies of the analyzed molecules appear as a series of bands in the Raman spectrum. These bands, detected by the spectrometer, correspond to the energy difference between incident and scattered light. The technique is applicable to solid, liquid, and gaseous samples, generally without extensive preparation, and can be performed on minimal sample volumes.

More than 25 Raman-based techniques have been described and are currently in use [17]. The principal modalities include:

¬ **Spontaneous Raman Spectroscopy** [17]

Conventional (or spontaneous) Raman spectroscopy represents the classical form based on inelastic light scattering. This is intrinsically an incoherent process: the scattered photons do not exhibit a deterministic phase relationship with the incident field. Consequently, the signal intensity is typically weak, with scattering cross-sections substantially lower than those of elastic scattering. For low-concentration systems, this often necessitates higher excitation powers, longer integration times, or signal-enhancement strategies.

¬ **Enhanced Raman Techniques**

**Micro-Raman spectroscopy (confocal Raman microscopy)** [21,22].

This approach employs a focused optical system to acquire Raman spectra from microscopic areas (typically on the micron scale), without plasmonic amplification. It is widely used for the analysis of biological tissues, cells, and material microstructures. A key advantage is that it does not require prolonged culture steps, enabling the analysis of isolated cells directly from blood, urine, or other biological fluids.

**Surface-Enhanced Raman Spectroscopy (SERS)** [23,24,25,26,27].

SERS exploits metallic nanostructures to amplify the Raman signal. In 1974, Martin Fleischmann first reported a marked enhancement of the Raman signal while studying pyridine adsorbed on a roughened silver electrode. Signal amplification is primarily attributed to two mechanisms: (I) electromagnetic enhancement, resulting from localized surface plasmons that concentrate the electric field into “hot spots” (with enhancement factors up to 10^8^–10^11^), and (II) chemical enhancement, related to charge transfer between the metal surface and the molecule (10^2^–10^3^). Owing to its high sensitivity, SERS is currently applied to the detection of explosives, nucleic acids, environmental pollutants, and pathogens.

**Tip-Enhanced Raman Spectroscopy (TERS)** [28].

TERS combines Raman spectroscopy with scanning probe microscopy (AFM or STM), using a plasmonic metallic tip to enhance both spatial resolution and Raman signal intensity at the nanoscale.

**Ultraviolet (UV) Raman Spectroscopy** [29,30].

This technique employs UV laser excitation to reduce background fluorescence and enhance Raman efficiency through resonance effects. It is particularly useful for the analysis of organic compounds and biomolecules, including proteins and peptides, and has applications in safety screening and pathogen identification. Ultraviolet resonance Raman (UVRR) spectroscopy typically analyzes bacterial samples in bulk, exposing a substantial biomass to UV light to mitigate photothermal damage; therefore, a preliminary culture step is often required. A major advantage of UVRR is the resonance enhancement of nucleic acid and aromatic amino acid signals, leading to improved signal-to-noise ratios.

¬ **Coherent Raman Spectroscopy**

**Coherent Anti-Stokes Raman Spectroscopy (CARS)** [31].

CARS is a nonlinear, high-sensitivity technique employing two or more laser beams (commonly referred to as pump and Stokes beams) to generate a coherent anti-Stokes signal.

**Stimulated Raman Scattering (SRS)** [31].

SRS is a nonlinear technique enabling rapid and quantitative chemical imaging. Unlike CARS, it does not exhibit the non-resonant background that can complicate signal interpretation in other coherent methods.

Despite the availability of multiple advanced modalities, conventional Raman spectroscopy remains the most widely used and accessible approach in clinical and laboratory practice.

## 4. Results

In the specific context of sepsis, Raman spectroscopy has been employed not only for the rapid screening of pathogens but also for the investigation of the host response. In particular, several studies have focused on the analysis of circulating immune cells, highlighting morphological and biochemical alterations associated with the septic state.

¬ **Application to immune cells**

The effectiveness of Raman spectroscopy in the diagnosis of sepsis lies in its ability to detect in real time variations in the molecular profile of immune cells and in the concentration of specific biomarkers during the septic event.

- **In vitro study in the field of nanobiotechnology and biomedical diagnostics**

Ying Wang et al. [32] employed surface-enhanced Raman scattering (SERS) for the simultaneous detection of the sepsis biomarkers interleukin-6 (IL-6) and procalcitonin (PCT). They developed a sandwich-type SERS platform consisting of multi-site boronic acid-functionalized magnetic nanomaterials (MB-MNPs) and interference-free antibody-modified probes. Initially, MB-MNPs enabled rapid biomarker enrichment; subsequently, high-sensitivity probes, exploiting antibody specificity, recognized IL-6 and PCT bound to the MB-MNPs. Analysis was performed on human serum samples (up to 10 µL) using SERS detection at 785 nm. The method demonstrated very high sensitivity, with extremely low limits of detection (0.584 and 2.99 pg/mL for Interleukin-6 and Procalcitonin, respectively), along with the added advantage of enabling the simultaneous quantification of multiple biomarkers. Quantitative biomarker analysis was then performed using a Raman spectrometer.

Anh H. Nguyen et al. [33] proposed a plasmonic mesoporous model for sepsis diagnosis. In this system, cysteine residues were reduced to active thiol groups that interacted with gold-coated magnetic nanoparticles, therefore enhancing the SERS signal and enabling magnetic separation. Based on immunoassay principles, this approach allowed triplex quantification of sepsis-specific biomarkers: PCT, C-reactive protein (CRP), and soluble triggering receptor expressed on myeloid cells-1 (sTREM-1). The limits of detection were 27 pM, 103 pM, and 78 pM for C-reactive protein (CRP), Procalcitonin (PCT), and sTREM-1 (soluble triggering receptor expressed on myeloid cells-1), respectively. Recombinant biomarkers, at concentrations ranging from 1 pM to 1 nM, were spiked into 1 mL of commercially available human serum (H4522, Sigma) to simulate real serum samples. SERS measurements were performed using an excitation wavelength of 785 nm.

Two additional studies by Xiaomei Wang et al. [34] and Ying Wang et al. [35]. further demonstrated the use of electromagnetically enhanced SERS systems for the rapid and sensitive quantification of sepsis biomarkers, specifically IL-6 and PCT (in the former study) and IL-6 alone (in the latter). In the first case, the limits of detection were 0.54 pg mL^−1^ and 0.042 ng mL^−1^ for Interleukin-6 (IL-6) and Procalcitonin (PCT), respectively. In the second study, a similarly low limit of detection (0.453 pg mL^−1^) was confirmed for Interleukin-6 (IL-6). As in the previous case, SERS measurements were performed using an excitation wavelength of 785 nm.

Olaetxea et al. [36] aimed to evaluate the applicability of Raman spectroscopy for the quantitative determination of lactate (in the concentration range 0–20 mM) and pH (7.0–7.5) in phosphate-buffered saline (PBS) solutions and in blood from domestic pigs stored at 4 °C. For the analysis, a Raman spectrometer equipped with a 785 nm excitation laser was employed. The scattered light was collected via an optical fiber and focused onto a monochromator. A filtering step was applied to remove background fluorescence, followed by baseline correction. Quantitative analysis was subsequently performed using advanced chemometric methods, specifically Partial Least Squares (PLS) regression.

Raman spectroscopy, combined with chemometric analysis (PLS), enabled accurate quantification of both lactate and pH in PBS solutions and in blood samples. The mean prediction error for lactate in blood samples was approximately 0.3 mM, while for pH it was 0.08 units, indicating good analytical performance within clinically relevant ranges.

Despite these promising results, the study presents several important limitations. First, the number of analyzed samples was limited (16 for pH and 12 for lactate), thereby reducing the statistical robustness of the developed models. This limited dataset also increases the risk of overfitting, with models potentially overly tailored to the training data and thus less generalizable. Another limitation concerns the lactate concentration range in the animal blood samples, which was already elevated (approximately 8.72 mM) due to procedural stress, restricting the analysis to values above normal human physiological levels. The authors therefore emphasize the need to substantially expand the experimental dataset in order to improve statistical validity and to develop more robust and clinically translatable predictive models (Table 2).

- **Preclinical studies conducted in animal models**

Osadare et al. [37] compared, by means of Raman spectroscopy, the molecular changes occurring in splenocytes from mice subjected to sepsis and hyperinflammatory endotoxemia. This is a preclinical experimental investigation conducted in an animal model, employing a biomolecular analytical approach based on Raman spectroscopy to comparatively characterize two distinct systemic inflammatory conditions. In this study, the differential Raman spectrum of septic mice (obtained by subtracting the mean Raman spectrum of septic mice from that of control mice) was compared with the differential Raman spectrum of endotoxemic mice (obtained by subtracting the mean Raman spectrum of endotoxemic mice from that of control mice). The spectral difference observed in septic mice was approximately one order of magnitude greater than that observed in endotoxemic mice. Measurements were performed 24 h after the endotoxin or infectious insult, during the acute inflammatory phase. The animals were sacrificed and the spleens were harvested for the isolation of splenocytes and T lymphocytes using magnetic separation. The cells were subsequently fixed and analyzed by Raman spectroscopy at an excitation wavelength of 532 nm to assess molecular differences between the experimental conditions. The limitation of the study is the small sample size of mice.

The most prominent alterations were detected in the DNA-associated spectral region of splenocytes, with less marked changes in protein and lipid bands. The disappearance of key amino acid vibrational signals in septic animals may be attributable to protein reorganization within activated T lymphocytes. Conversely, in healthy cells, elevated lipid levels appear to be linked to the preservation of vital physiological functions. While clear discrimination was achieved between septic and control mice, spectral discrimination between septic and endotoxemic mice proved more challenging.

Meiyan Wu et al. [38] investigated mitochondrial redox status in vivo in mitochondria isolated from the gastrocnemius muscle of male mice. Using resonance Raman spectroscopy (RRS), spectra were acquired at 30 min, 1, 2, 4, and 6 h after cecal ligation and puncture (CLP) in septic mice. This longitudinal preclinical study aimed to evaluate the diagnostic and prognostic potential of mitochondrial redox states. Blood lactate levels were used as a predictor of sepsis. At 2 h post-CLP, a significant fluctuation in the peak area corresponding to the reduced mitochondrial Raman fraction was observed preceding the significant increase in lactate levels, which occurred at 12 h post-CLP. In addition to providing earlier detection than lactate elevation, mitochondrial redox assessment demonstrated superior prognostic accuracy: a threshold value of 1.059 predicted septic outcomes with 80% sensitivity and 90% specificity. Spectra were acquired using 532 nm excitation. An increase in intensity near the 750 cm^−1^ redox peak was observed in the RRS spectrum of the reduced mitochondrial form compared with the partially oxidized form in controls. This increase was attributed to reduced concentrations of NAD^+^ and oxidized glutathione (GSSG). The extraction of Raman bands from oxidized cytochromes to assess mitochondrial redox states presents challenges, as cytochrome c and b are difficult to distinguish, and the 532 nm laser penetrates only up to 2 mm, limiting in vivo information. Furthermore, the assessment requires the surgical exposure of muscle, reducing clinical feasibility. Nevertheless, this study provides a promising insight: non-invasive resonance Raman spectroscopy (RRS) could offer a novel biomarker to monitor mitochondrial function and predict sepsis outcomes (Table 3).

- **Prospective study in human models**

In contrast, Ramoij et al. [39] conducted a prospective, nonrandomized, single-center observational phase II study to evaluate the diagnostic accuracy of Raman spectroscopy-based leukocyte profiling in differentiating inflammation, infection, and sepsis in hospitalized patients. Compared with conventional biomarker information alone, the addition of Raman-derived data increased diagnostic accuracy for infection detection by 10% (to 93%) and for sepsis detection by 18% (to 92%). Leukocytes from septic patients exhibited distinct Raman spectral features compared with those from patients with infection alone, reflecting a sepsis-specific immune phenotype. Spectroscopic analyses were performed within 8 h of blood sampling, during the acute inflammatory phase. More prominent nucleic acid bands (1582 cm^−1^ and 778 cm^−1^) were observed in leukocytes from patients with sterile inflammation compared with those from patients with infection/sepsis. Raman bands around 1664 cm^−1^ and 1434 cm^−1^ indicated altered protein and lipid composition. This study demonstrates that spectroscopic assessment of leukocyte activation status may be used either independently for sepsis diagnosis or as an adjunct to conventional biomarkers. All 61 patients enrolled in the study met at least one SIRS criterion, including (1) fever >38.0 °C or hypothermia <36.0 °C, (2) tachycardia >90 beats/min, (3) tachypnea >20 breaths/min, or (4) leukocytosis >12 × 10^9^/L or leukopenia <4 × 10^9^/L. An aliquot of 500 µL of EDTA-anticoagulated blood was used for Raman spectroscopy analysis. Spectroscopic measurements were performed at the single-cell level using a commercial vertical micro-Raman system (CRM 300; WITec GmbH, Ulm, Germany) equipped with a 785 nm diode laser.

Despite its promising results, the study presents several structural limitations that currently hinder its immediate clinical applicability. The relatively small sample size (61 patients) and the single-center design (Jena, Germany) limit the statistical robustness and generalizability of the findings. Moreover, an intrinsic limitation of Raman Spectroscopy lies in the complexity of spectral interpretation: although spectral variations can be detected, attributing these changes to specific proteins or genes remains highly challenging.

Additionally, since neutrophils represent the most abundant leukocyte population, the Raman signal is predominantly driven by these cells. However, sepsis also critically involves monocytes, which accounted for less than 8% of the analyzed cells in this study. This underrepresentation may obscure important aspects of the immune response.

Finally, although the authors highlight the potential applicability of this approach within the clinical “golden hour,” the current workflow still requires multiple sample preparation steps (including lysis, fixation, and spin-coating) as well as data analysis procedures that would need further automation to be compatible with the time constraints of an intensive care setting (Table 4).

¬ **Application to pathogens.**

The Raman-based approach enables accurate, rapid, and potentially blood-culture-independent detection of pathogens in blood.

- **Raman spectroscopy for the assessment of pathogen susceptibility to antimicrobial agents.**

In a European study, the sensitivity of Raman spectroscopy, as evaluated on positive blood culture samples after 5 h of incubation, was comparable to the VITEK 2 system for the identification of 133 strains, while offering a significant advantage in diagnostic turnaround time. The study reported the use of the VITEK 2 Compact system (bioMérieux, Marcy-l’Étoile, France).

The study was designed in two sequential phases: an initial proof-of-concept phase conducted on standardized bacterial cultures, followed by a proof-of-principle (investigative) phase using simulated blood culture samples. A total of 133 bacterial strains (67 susceptible and 66 resistant) were included.

For sample preparation, bacterial strains were incubated in tryptic soy broth (TSB) for 5 h in the presence or absence of different antibiotic concentrations. To simulate clinical conditions, negative blood culture bottles (after 120 h of incubation) were spiked with approximately 500 CFU of bacterial isolates and incubated overnight. Bacteria were subsequently isolated from blood samples using serum separator tubes and red blood cell lysis.

Raman measurements were performed using a 785 nm diode laser. Results were compared with established reference methods, including the VITEK^®^ 2 system and broth dilution assays [40].

Despite the promising findings, several limitations were identified. First, further optimization is required to achieve the level of robustness necessary for routine clinical diagnostic implementation, comparable to established platforms such as VITEK^®^ 2. Second, while full concordance (100%) was observed for antibiotic-susceptible strains, agreement for resistant strains was lower (68% compared to VITEK^®^ 2), likely due to inter-laboratory variability in inoculum preparation and antibiotic handling. Additionally, the bacterial isolation procedure from blood cultures remains labor-intensive, highlighting the need for simplification and automation. Challenges were also noted in the interpretation of “intermediate” susceptibility results, particularly near clinical breakpoints defined by CLSI, where discrepancies with conventional methods were observed. Finally, although the proof-of-principle phase using blood culture samples yielded encouraging results, it was conducted on a limited and representative strain set, restricting the generalizability of the findings.

A similar comparison was performed for antibiotic susceptibility testing using the SERS-AST protocol, which demonstrated high concordance with the VITEK 2 diagnostic system (96% for Gram-positive bacteria and 97% for Gram-negative bacteria), again within a 5 h timeframe. The process begins with the collection of infected blood. Hemoglobin exhibits significant spectral interference and would completely obscure the signals arising from bacteria. For this reason, the sample undergoes an extensive purification step, including lysis and washing.

After an incubation period of 2–3 h, the bacteria are deposited onto a specialized substrate functionalized with silver nanoparticles. The sample is then irradiated with a 632.8 nm laser (red light). The scientists tested 164 samples from real patients. The result is a method that not only determines whether a bacterium is sensitive or resistant but does so at a controlled bacterial concentration (up to 3 × 10^9^ CFU/mL) [41]. In this case as well, several critical issues emerge. Certain antibiotics, such as levofloxacin, exhibit slower mechanisms of action; consequently, the results show lower concordance, suggesting that the incubation time should be extended to at least three hours to obtain reliable data.

Additional challenges are associated with specific bacterial species. In 36% of *Klebsiella pneumoniae* isolates, a discrepancy between SERS and VITEK 2 results was observed, likely due to the organism’s polysaccharide capsule, which hinders the secretion of metabolites required for SERS signal generation. Conversely, Acinetobacter baumannii exhibited low membrane permeability, necessitating additional treatments to enhance signal detection.

The study also highlights several important exclusions: bacteria such as Pseudomonas aeruginosa were not analyzed due to their strong intrinsic fluorescence, which interferes with the SERS signal, and fungal infections, such as Candida albicans, were not addressed at this stage.

Raman spectroscopy has also been applied to vancomycin susceptibility testing in Enterococcus faecalis and Enterococcus faecium during exponential growth following exposure to 10 μg/mL vancomycin, achieving a positive predictive value (PPV) of 93% and a negative predictive value (NPV) of 96%. Regarding sample preparation, bacterial cultures were grown and subsequently subjected to a pre-incubation phase until reaching the exponential growth stage, which represents optimal conditions for analysis. Different culture media were employed depending on the species: CASO medium for Enterococcus faecalis and LB medium for Enterococcus faecium. Bacterial suspensions were standardized to a concentration of approximately 10^8^ cells per milliliter, ensuring controlled and reproducible experimental conditions. The analysis was performed using the DEP-Raman technique, with sample excitation achieved using a 532 nm laser [42].

Despite the promising results, the study presents several significant limitations. First, it constitutes a proof-of-concept based on a limited number of biological replicates (9 for *E. faecalis* and 6 for *E. faecium*), which restricts the generalizability of the findings. Furthermore, the diversity of the analyzed strains remains limited; inclusion of strains with different resistance mechanisms and varying minimum inhibitory concentration (MIC) values will be necessary to improve the robustness of the model. Another limitation is the lack of a substantial number of real clinical samples, which are essential to assess the method’s performance under practical, real-world conditions. Finally, the model’s specificity for *E. faecium* was approximately 77%, indicating that a considerable proportion of susceptible strains are still not correctly classified.

Comparable findings have been reported in another study in which Raman spectroscopy detected differential pathogen responses to vancomycin concentrations within only 90 min. In this study, employing a similar methodology, 11 independent batches, corresponding to distinct biological replicates, were analyzed to ensure greater reliability of the results. Overall, thousands of Raman spectra were collected, encompassing both control samples and antibiotic-treated samples, and acquired at multiple time points (0, 30, 60, and 90 min). This time-resolved approach enabled dynamic monitoring of treatment-induced molecular changes.

Regarding bacterial parameters, although an exact number of colony-forming units (CFU) per sample was not reported, the study indicates that the initial culture had an optical density (OD) of 0.1. Antibiotic treatment was initiated when the culture reached an OD of 0.6–0.8, corresponding to the exponential growth phase.

The analysis was carried out using micro-Raman spectroscopy with a WITec CRM 300 system, with signal excitation provided by a 532 nm Nd:YAG laser [43].

In 2021, a study evaluated a Raman-assisted antibiotic susceptibility test (FRAST) applied to nine infectious urine samples and three sepsis samples. Results were consistent with those obtained using MALDI-TOF mass spectrometry and conventional AST. In the preclinical phase, 3200 treatments with 38 different antibiotics were assessed. In single-cell Raman spectra (SCRS), antimicrobial resistance was reflected by a distinct Raman band in the 2000–2300 cm^−1^ region, attributable to active incorporation of deuterium from heavy water (D_2_O).

The underlying principle of the methodology is the incorporation of deuterium derived from heavy water (D_2_O) into the biomolecules of metabolically active bacteria. In the presence of antibiotics, resistant bacteria continue to metabolize and therefore incorporate deuterium, forming carbon deuterium (C-D) bonds that can be detected by Raman spectroscopy. In contrast, in susceptible bacteria, metabolic activity is inhibited and the deuterium signal does not emerge. This enables rapid and direct discrimination between resistant and susceptible strains.

The study included both standard quality control strains, comprising Gram-negative and Gram-positive bacteria, and real clinical samples, including urine samples from urinary tract infections and blood samples associated with sepsis. Control strains were cultured in Mueller–Hinton broth, whereas urine samples, due to their high bacterial load, were analyzed directly without prior cultivation. Blood samples, on the other hand, required an incubation step in aerobic culture bottles until positivity, typically after approximately 18 h.

For susceptibility testing, bacterial suspensions were standardized to a concentration of approximately 5 × 10^5^ CFU/mL. The analysis was performed using single-cell confocal micro-Raman spectroscopy (SCRS), employing a laser source with an excitation wavelength of 532 nm [44].

Despite the promising results, particularly considering the ability to analyze urine samples directly without any preprocessing, the method still presents several limitations. In particular, signal saturation represents a critical issue: if deuterium is added simultaneously with the antibiotic, bacteria may incorporate it before the drug exerts its effect, potentially leading to false-positive resistance results. Furthermore, blood samples still require a preliminary cultivation step of approximately 18 h, bringing the total analysis time to around 21 h. These findings suggest promising future applications in point-of-care diagnostic devices.

Kang et al. evaluated the diagnostic performance of an SERS-based approach combined with deep learning [45], analyzing approximately 12,000 spectra. From blood samples of patients with positive blood cultures, detection accuracy reached 98.68% for pathogenic bacteria and 99.85% for carbapenem-resistant *Klebsiella pneumoniae*. Differential centrifugation outperformed erythrocyte lysis for bacterial isolation from blood. Convolutional neural networks (CNNs) demonstrated substantial potential for identifying prevalent pathogens and their drug-resistant strains. A total of 130 positive blood culture bottles, collected between June and September 2023, were analyzed. The original samples were derived from routine clinical blood cultures that had already tested positive. To establish the reference “gold standard”, bacteria were subcultured on blood agar plates to obtain isolated colonies, which were subsequently identified using MALDI-TOF mass spectrometry. However, the aim of the proposed method is the direct identification from the positive blood culture bottle, without waiting for colony growth on agar plates. Measurements were performed using a laser source with an excitation wavelength of 785 nm, employing the SERS technique. In all samples considered for SERS analysis, the bacterial concentration exceeded 10^9^ CFU/mL [46].

Although the study reports high accuracy (up to 99.85% for the identification of carbapenem resistance), several intrinsic limitations can be identified, either explicitly stated or inferred. The study excluded samples with mixed (polymicrobial) infections, focusing exclusively on single-strain isolates. Furthermore, the method still requires the blood culture bottle to become positive and therefore does not completely eliminate the need for incubation. Finally, for resistance analysis (CRKP vs. CSKP), the number of cases was relatively limited, with only 8 cases of carbapenem-resistant *Klebsiella pneumoniae* (Table 5).

- **Raman spectroscopy for pathogen identification**

In an in vivo murine study based on SERS analysis of blood serum, diagnostic sensitivity of 87.5% and specificity of 94.7% were achieved for the detection of Trichinella spiralis infection, using multivariate statistical techniques such as principal component analysis (PCA) and linear discriminant analysis (LDA). Serum samples from rats were analyzed. Specifically, blood was collected from the tail vein of female Sprague-Dawley rats.

Initially, 40 animals were included; however, due to mortality occurring during anesthesia, the final study comprised 35 samples collected at 28 days post-infection: 19 in the control (non-infected) group and 16 in the infected group. The samples were not cultured in the microbiological sense (as would be done for bacteria). Since the pathogen is a parasite (Trichinella spiralis), infection was induced by orally administering 3500 muscle larvae (ML) to the rats. SERS analysis was performed using an excitation wavelength of 785 nm.

The study was conducted on a relatively small sample of rats. The authors themselves identify, as a next step, the need to validate the method in additional hosts, including swine and humans [47].

Another study combining SERS and deep learning for direct, culture-free sepsis diagnosis from blood trained on over 650 samples and validated on an independent cohort achieved 99.67% accuracy for binary sepsis classification and 98.84% accuracy for six-class pathogen identification. Residual misclassifications primarily involved control samples and *Escherichia coli*, as well as certain Gram-negative species. Raman spectra were acquired using SERS with an excitation wavelength of 785 nm. Whole blood samples were collected from patients with clinically diagnosed sepsis and from control (negative) subjects using a culture-free approach. However, the blood samples obtained from patients were initially inoculated and incubated in enrichment broth bottles of the automated BD BACTEC blood culture system, from which aliquots were subsequently extracted for SERS analysis. A total of 723 blood samples were analyzed, using 3 μL of blood per measurement [48].

Overall, this methodology represents a rapid and potentially point-of-care applicable solution with tangible prospects for improving early sepsis diagnosis and clinical management. The high sensitivity of SERS was further demonstrated in a preclinical study using MoS_2_-Au nanoparticle substrates, which enabled the detection of *Staphylococcus aureus* and *Escherichia coli* down to 10^2^ CFU/mL. The analyzed samples were divided into two categories: hazardous and toxic dye molecules (Rhodamine B and N719) prepared in ethanol solutions, and biological samples containing the pathogenic bacteria *Staphylococcus aureus* and *Escherichia coli*.

The bacterial samples were cultured. Specifically, *S. aureus* and *E. coli* cultures were prepared by inoculating the bacteria into a liquid medium (LB broth) and incubating them overnight at 37 °C. Experiments on bacterial samples covered concentrations ranging from a maximum of 10^8^ CFU/mL, followed by serial dilutions (10^6^, 10^4^), down to a minimum detectable concentration of 10^2^ CFU/mL, at which successful detection was still achieved.

The technique employed was SERS with a 532 nm excitation wavelength (coupled to a 50× objective). It was observed that at very high concentrations (10^8^ CFU/mL), the excessive bacterial load physically covers the substrate, limiting the effectiveness of the SERS “hot spots” and resulting in highly noisy spectra [49]. Ultraviolet resonance Raman (UVRR) spectroscopy achieved 92% accuracy in classifying Klebsiella species in a study of 24 clinical isolates, including *Escherichia coli*, *Klebsiella pneumoniae*, and *Klebsiella oxytoca*, using single-cell Raman microspectroscopy with 532 nm excitation.

The spectra were acquired with two spectroscopic approaches: UV resonance Raman spectroscopy (UVRR) and single-cell Raman microspectroscopy.

Identification of the isolates was performed using the Vitek-2 system and they were identified as 15 strains of *Klebsiella pneumoniae*, six strains of *Escherichia coli,* and three strains of *Klebsiella oxytoca*. For Raman measurements, bacteria were cultured from frozen stock on nutrient agar and incubated overnight at 37 °C. A small amount of biomass was then transferred to nutrient broth and incubated at 37 °C with shaking at 120 rpm.

For single-cell Raman microspectroscopy (SC-RMS), cells were cultured overnight in 5 mL of nutrient broth. Samples were prepared by adding 100 μL of bacterial culture to 900 μL of distilled water. Finally, 10 μL of the sample was deposited in 1 μL drops on a nickel foil disc and allowed to dry at room temperature for 15–60 min. Before UV-Resonance Raman (UVRR) measurements, bacteria were cultured for 1 h in 20 mL of nutrient broth to reach the exponential growth phase. Three 1.5 mL replicates of the inoculum each were heat-inactivated at 99 °C for 5 min, followed by three consecutive washes. The final pellet was resuspended in 30 μL of distilled water and allowed to dry on a fused silica slide at room temperature for 1 h.

UVRR spectroscopy yielded better accuracy for differentiation compared with SC-RMS with 532 nm excitation. This is because the resonance effect of nucleic acids in this excitation wavelength could capture the differences present in the genomes of the isolates.

The study describes Raman spectroscopy as a promising method because it enables the rapid, label-free differentiation of *E. coli* and *Klebsiella*. However, these results could be affected by the limited generalizability (the study analyzed a relatively small number of clinical isolates), the intra-species variability that impacts Raman spectroscopy, the lack of extensive clinical validation, and the absence of other microorganisms contaminating the sample. An additional limitation is the failure to specify the number of colony-forming units, as the quantity of bacteria can affect the intensity of the Raman spectrum and therefore the reproducibility of the results: the main focus of the study is on bacterial differentiation, not on quantifying the bacterial load [50].

Positive identification results were also reported through the combination of magnetic separation using M13 bacteriophage-coated beads and micro-Raman spectroscopy. This approach required approximately 6 h for analysis and achieved a detection limit of 10 colony-forming units in 7 mL of blood. Phage clones were selected that bind to the surface of *P. aeuriginosa*, *E. coli* and *S. aureus*. Subsequently, commercial magnetic microspheres were functionalized with phage clones via covalent binding and used for the capture and concentration of pathogens from the blood [51].

In another study, Raman spectroscopy identified strains from cultured colonies, with 2.8% and 7% of strains misidentified using the support vector machine algorithm based on centered and uncentered principal component analyses, respectively. The most problematic species was Proteus vulgaris, whose spectra were frequently misclassified as *Escherichia coli*. Suboptimal results were also reported for *Candida tropicalis*, *Enterococcus faecalis*, *Staphylococcus warneri*, *Staphylococcus lugdunensis*, and *Streptococcus oralis*.

Pyoverdin-associated Pseudomonas aeruginosa peaks include vibrations at 715,830, 1355, 1488, and 1611 cm^−1^. In staphylococcal species, we can detect carotenoid pigments visible at wavenumbers of 1110, 1160 and 1525 cm^−1^, corresponding to the vibrations CC-(CH_3_), =CC= and -C=C. However, other factors contribute to the variance of the spectra, including but not limited to virulence factors, colony age and metabolic processes, and overall intraspecific biological variability [52].

Diagnostic performance may be further enhanced through the integration of Raman tweezers with microfluidic chambers, providing a robust optofluidic platform for single-cell manipulation and analysis via optical microscopy and Raman spectroscopy. This was demonstrated in a study isolating *Escherichia coli* and assessing cefotaxime resistance.

Optical micromanipulation in a microfluidic chamber chip was tested by transferring individual bacteria into the chambers. Cells in the chambers were exposed to the antibiotic cefotaxime, and changes were observed using time-lapse microscopy. Separately, laser tweezers Raman spectroscopy (LTRS) was used in a different microcamera chip to manipulate and analyze single *E. coli* cells treated with cefotaxime. Additionally, conventional Raman microspectroscopic measurements of *E. coli* cells were performed using a microcamera. The optical trap and a microcamera based on an optofluidic system allowed us to effectively isolate individual *E. coli* bacterial cells and observe the morphological changes induced by the cephalosporin antibiotic cefotaxime. These measurements were performed with Raman excitation and trapping wavelength at 785 nm. Raman microspectroscopy of bacterial samples at 532 nm provided us with spectra complementary to measurements at 785 nm. The study by Pilàt et al. [53] provides encouraging insights into the use of microfluidics and Raman spectroscopy; however, the lack of clinical data, the small sample size, and the favorable but non-replicable experimental conditions in real-world settings limit the validity and generalizability of the results

An acoustofluidic technique combined with SERS was validated for detecting varying concentrations of Escherichia coli (1.75 × 10^5^ CFU/mL in under one hour). Bacteria were labeled with SERS nanotags and flowed through a silicon microfluidic channel. Acoustic waves generated by a piezoelectric transducer separated larger labeled bacteria from unbound nanotags, concentrating them at the center of the channel, where a focused laser measured Raman signals for quantitative analysis [54]. Additionally, Raman tweezers could detect the presence of microbes in human serum and characterize them, which could reduce the time required to identify a pathogen to <10 min after receiving a clinical sample. Among major nosocomial bloodstream pathogens, methicillin-resistant *Staphylococcus aureus* (MRSA) has also been investigated. Single-cell Raman analysis with 532 nm excitation or 785 nm excitation directly on Petri dishes discriminated MRSA from methicillin-susceptible strains (MSSA) with 87.5% accuracy. The main disadvantage of single-cell analysis is the high heterogeneity in the sample due to cell–cell differences in growth stage and metabolism. Single-cell analysis with excitation at 532 nm has been shown to be the best methodology, as it provides information on the overall biochemical composition of the bacteria. In fact, the discrimination between MRSA and MSSA when using 785 nm excitation directly on bacterial colonies is based on the staphyloxanthin bands that show a higher intensity in MRSA strains. However, staphyloxanthin is a cellular component not directly related to antibiotic resistance [55]. Streptococcus pneumoniae, a frequent cause of severe septic syndromes with high mortality, was identified with balanced accuracy of approximately 70% when differentiated from *Streptococcus dysgalactiae*, *Streptococcus pyogenes*, *Streptococcus thermophilus*, and *Streptococcus sanguinis*, indicating theoretical discriminative feasibility but still suboptimal accuracy, as a larger dataset is needed to build reliable classification models. The *S. pneumonia* has a higher Raman band at 720 cm^−1^ (band assigned to adenine, the N+(CH 3) 3 head group of lipids and choline), at 872 cm^−1^ (band assigned to another N+(CH 3) 3), at 856 cm^−1^ (probably due to COC stretching vibrations and CC deformation vibrations in glycosidic compounds), at 956 cm^−1^ (attributed to COC stretching vibrations—α-D-1,4 glycosidic bond, α-D-1,6 glycosidic bond—in polysaccharides as well as in the amino acids tryptophan and valine), at 2856 cm^−1^ and 2880 cm^−1^ (assigned to CH2 stretching vibrations in lipids). *S. pneumoniae* has a lower relative Raman intensity in the range of 1336 cm^−1^ to 1376 cm^−1^ than other streptococci. This region is assigned to nucleic acids (adenine, thymine, and guanine) and the amino acid tryptophan, as well as to CH deformation vibrations in proteins and polysaccharides.

Choline incorporated into the pneumococcal cell wall may be responsible for these spectral differences [56]. In this study, an analysis was performed on pure bacterial suspensions, not on complex clinical samples (such as blood, sputum, or swabs); therefore, performance under real-world conditions has yet to be validated.

*Klebsiella pneumoniae* and *Acinetobacter baumannii*, two clinically significant Gram-negative pathogens in sepsis, were detected with a limit of detection of 10 cells/mL and a total analysis time of 1.5 h. In this study, a dual-function SERS aptasensor based on a dual recognition strategy was developed for the multiplex detection of KP and AB in food and clinical samples. The SPION-PEI-Au-Van nanocomposite (used as a universal capture probe for bacteria) achieved capture efficiencies of 78.1% for *Klebsiella pneumoniae* and 75.2% for *Acinetobacter baumannii* [57]. The limited capture efficiency of this test may be related to the food-based nature of the sample and the low concentration of the pathogen.

Even greater sensitivity has been demonstrated in candidemia. SERS enabled detection of *Candida albicans*, *Candida glabrata*, *Candida tropicalis*, *Candida auris*, *Candida haemulonii*, *Candida dubliniensis*, and *Candida parapsilosis* in 7.5 mL of whole blood at clinically relevant concentrations as low as 2 CFU/mL, with results obtained within 4–5 h.

A study was designed that combines antibody-modified magnetic microspheres for pathogen isolation, SERS-encoded magnetic tags for multiplex detection, and fluorescence microscopy for rapid pre-screening. High specificity was demonstrated, with minimal cross-reactivity with bacterial controls [58]. Despite the promising results, it is important to consider the lack of clinical variability and the absence of real physiological conditions, which may overestimate the study’s efficacy compared to a real clinical setting.

Also, Pezzotti et al. [59] provide a detailed analysis of Raman spectroscopy applied to the species most commonly responsible for oral candidiasis, including *Candida albicans*, *Candida glabrata*, *Candida tropicalis*, and *Candida krusei*. The authors collected oral samples containing the different Candida species and subjected them to microbiological culture to isolate individual species. The resulting colonies were analyzed using a Raman barcode approach, in which spectrally deconvoluted Raman sub-bands are converted into barcode-like patterns, allowing for a structured and simplified interpretation of spectral information. For each colony, multiple high-resolution Raman spectra were acquired, and the spectral data were subsequently processed using chemometric methods. All analyses were performed on isolated colonies rather than on complex biological fluids, meaning that the study does not capture the full complexity of raw clinical samples. Nonetheless, this strategy could enable in situ, near real-time identification of different Candida species. However, spectral overlap in polymicrobial infections may complicate accurate recognition, and further standardization of the method is required for direct application to complex biological specimens. A LabRAM HR800 (Horiba/Jobin-Yvon) confocal Raman spectrometer operating at 532 nm was employed for the analyses.

Primary urine samples from patients with suspected urinary tract infection were analyzed by Aubrechtová Dragounová K et al. [60]. using Raman spectroscopy for rapid bacterial identification. Eighty-five strains were included, encompassing species such as *Escherichia coli*, *Klebsiella pneumoniae*, *Pseudomonas aeruginosa*, *Enterococcus faecalis*, *Enterobacter cloacae*, *Proteus mirabilis*, *Streptococcus pyogenes*, *Staphylococcus aureus*, *Providencia rettgeri*, *Citrobacter koseri*, *Morganella morganii*, *Acinetobacter ursingii*, and *Corynebacterium amycolatum*. Using a vertical micro-Raman system with 532 nm excitation, mixed Gram-positive/Gram-negative infections were initially differentiated from single-group infections, achieving 49% accuracy due to high sensitivity but low specificity for Gram-negative bacteria. Distinct spectral differences between Gram-positive and Gram-negative organisms were observed, particularly in the 2850 cm^−1^ region (more concentrated with lower variance in Gram-positive species) and around 1420 cm^−1^ (more abundant in Gram-positive bacteria). Overall classification accuracy reached approximately 75%. Spectral differences were observed between isolates grown on standard culture medium and bacteria of the same strain characterized directly by the patient. Therefore, an improvement in classification accuracy is expected with a larger database that also includes bacteria measured directly from the urine sample (Table 6).

## 5. Discussion

The etiological diagnosis of sepsis currently relies on direct microbiological techniques such as MALDI-TOF mass spectrometry and polymerase chain reaction (PCR).

MALDI-TOF (Matrix-Assisted Laser Desorption/Ionization Time-of-Flight) identifies microorganisms by analyzing their protein profiles. In this approach, the microbial sample (typically obtained from a positive blood culture) is ionized, and the resulting mass spectrum is compared with a reference database. Advantages include rapid turnaround (identification within 1–2 h), high accuracy for bacterial species, and the possibility of integrating antimicrobial resistance testing [61].

However, its use is limited to positive blood cultures. MALDI-TOF also encounters greater difficulty in generating high-quality spectra for fungal infections, necessitating standardization of culture media, extraction procedures, and sample preparation. A critical limitation is the lack of comprehensive and robust spectral databases for certain fungal species. These challenges largely stem from the greater structural complexity of yeast and mold cell walls compared with bacterial cell walls, which require additional protein extraction steps. Optimization has been achieved through formic acid/acetonitrile pretreatment extraction protocols [62].

Additional limitations include difficulties in identifying certain Gram-positive organisms (e.g., coagulase-negative staphylococci) and reduced performance in polymicrobial samples or specimens with low bacterial load. In polymicrobial infections, the resulting spectrum represents the sum of individual spectra from all species present, significantly reducing diagnostic resolution and highlighting the need for alternative molecular approaches [63].

Takuya Hosoda evaluated the application of whole genome sequencing (WGS), which enabled the identification of species not detected by MALDI-TOF in 13% of analyzed samples [63]. Direct detection methods such as PCR have also shown promise, demonstrating greater sensitivity and specificity than MALDI-TOF in diagnosing and discriminating against polymicrobial infections: PCR relies on pathogen-specific primers capable of simultaneously detecting multiple microorganisms within the same sample [64]. Nonetheless, limitations include high costs, risk of contamination-related false positives, and dependence on predefined panels that may not include all clinically relevant pathogens.

What fundamentally distinguishes Raman spectroscopy from traditional microbiological methods is not only its speed, but also the intrinsic nature of the information generated: a spectral fingerprint reflecting the metabolic, structural, and functional state of the analyzed sample. Raman spectroscopy enables acquisition of a direct molecular signature without the need for labeling or prior culture, providing rapid, high-content biochemical information, particularly in its surface-enhanced variant (SERS) and when integrated with deep learning algorithms.

In summary, while MALDI-TOF is the gold standard for rapid identification from positive blood cultures, its efficacy diminishes in fungal or polymicrobial infections. PCR provides high sensitivity for difficult-to-culture pathogens but remains constrained by high costs and the risk of false negatives for pathogens not included in predefined primer panels. Raman spectroscopy, although it still requires standardization and clinical validation on a large scale, is not limited by predefined panels or interference of human DNA during amplification and can detect pathogens not included among the targets of PCR.

The choice of the optimal technique depends on the clinical context, the microbiological suspicion and the additional information needed (Table 7).

In Raman spectroscopy, scattered light carries spectral information specific to the molecular composition of the analyzed region, including: proteins, polysaccharides, lipids, and nucleic acids in pathogen-specific configurations [65].

Streptococcus pneumoniae differs from other oral streptococci by exhibiting more intense bands at 720 cm^−1^ and 872 cm^−1^ attributed to adenine, N^+^(CH_3_)_3_ groups, and choline as well as increased intensity at 956 cm^−1^, associated with C–O–C vibrations in polysaccharides and amino acids. Conversely, lower intensity at 2936 cm^−1^ (protein CH_3_ stretching) reflects differences in protein content. Streptococcus pneumoniae displays a higher lipid-to-protein ratio, evidenced by stronger lipid- and polysaccharide-associated bands and weaker amino acid bands. The 872 cm^−1^ choline band serves as a distinctive marker [56].

Gram-positive bacteria generally produce more intense Raman bands associated with peptidoglycan components of the cell wall (e.g., 730–750 cm^−1^, 1450 cm^−1^), whereas Gram-negative organisms exhibit more pronounced signals corresponding to outer membrane lipids (e.g., 2850–2930 cm^−1^) [66].

For example, *Escherichia coli* is characterized by strong bands at 1004 cm^−1^ (phenylalanine), 1336 cm^−1^ (adenine), and 1450 cm^−1^ (lipid CH_2_), reflecting a defined lipid/protein profile [67]. *Staphylococcus aureus* exhibits prominent amide-associated bands (e.g., 1655 cm^−1^, amide I) and can be differentiated from methicillin-resistant strains (MRSA) through variations in amide II bands and nucleotide/protein ratios [68]. Phenazine pigment bands (1370–1380 cm^−1^) are diagnostically relevant for Pseudomonas aeruginosa [69].

These spectral differences reported in different studies are exploited for microbiological identification using machine learning classification models, multivariate analysis (PCA, LDA), and deep learning algorithms, giving accurate discrimination among genera, species, and strains, even directly from clinical samples or at the single-cell level.

As demonstrated in our findings, the clinical utility of Raman spectroscopy (RS) in sepsis can be categorized into four interconnected functional domains:

- Host Immune Response Profiling: Unlike traditional microbiology, RS captures the host’s “septic phenotype.” Studies on splenocytes and leukocytes [37,39] demonstrate that Raman spectroscopy can detect early biochemical alterations (such as nucleic acid bands at 1582 cm^−1^ and protein bands at 1664 cm^−1^) that precede systemic markers like lactate. This form of “metabolic staging” could enable a precision medicine approach to immunotherapy.
- Rapid Pathogen Identification: The technique exploits specific molecular vibrations, such as the adenine band at 720 cm^−1^ for *S. pneumoniae* [58] or phenazine-associated signals for *P. aeruginosa* [69]. Integration with deep learning approaches, particularly convolutional neural networks (CNNs), has increased diagnostic accuracy beyond 90% [45,48]. However, species with high phenotypic plasticity or thick capsules (e.g., *Klebsiella pneumoniae*) continue to pose significant spectral challenges.
- Accelerated Antimicrobial Susceptibility Testing (AST): By monitoring deuterium (D_2_O) incorporation [44] or employing Raman optical tweezers [55], it is possible to observe bacterial metabolic arrest in the presence of antibiotics within 1.5–3 h, surpassing the 24–48 h delay associated with conventional phenotypic methods such as VITEK 2.
- Multiplex Biomarker Detection: Surface-enhanced Raman spectroscopy (SERS) platforms have demonstrated the ability to simultaneously quantify PCT, IL-6, and CRP with femtomolar sensitivity [32,35]. This triple detection from a single 10 µL serum sample represents a generational advancement over traditional ELISA-based assays.

Despite these technical achievements, several critical limitations hinder clinical translation. First, the biological interpretability of Raman signatures remains incomplete. For instance, differentiation between MRSA and MSSA is often driven by staphyloxanthin pigments [57], which are not the direct molecular determinants of methicillin resistance. This may introduce a risk of false negatives under varying environmental conditions.

Furthermore, significant technical barriers also persist. Pigmented pathogens, such as *P. aeruginosa*, can emit strong fluorescence that overwhelms the Raman signal, often necessitating UV-Raman or Surface-Enhanced techniques to improve the signal-to-noise ratio. Additionally, requirements for sample purification (e.g., removal of hemoglobin) and prolonged incubation times for slow-acting antibiotics (e.g., levofloxacin) indicate that fully autonomous application within the “golden hour” is not yet feasible. Furthermore, the lack of SERS signal standardization affects nanostructure stability; even with improved AgNP/AAO substrates, inter-batch variability remains a concern for diagnostic certification (Table 8).

Another strength of Raman spectroscopy is its versatility in sample preparation. In many cases, biological material can be analyzed directly, minimizing preprocessing steps and reducing analysis time.

In recent years, several next-generation Raman variants have been developed. Among these, SERS significantly enhances signal intensity through nanostructured surfaces, improving sensitivity even at low analyte concentrations. Other variants include UV-Raman and the use of different excitation wavelengths, each offering specific advantages in terms of resolution and background silence. These characteristics position Raman spectroscopy as an ideal candidate for point-of-care applications, potentially integrable into emergency department diagnostic workflows.

This rapid and non-destructive technique can be applied to multiple biological fluids relevant to sepsis. Experimental studies have demonstrated pathogen identification in ascitic fluid, contributing to the rapid diagnosis of bacterial peritonitis [70]. Similarly, urine analysis via Raman spectroscopy has shown positive results for urinary tract infection screening [69], while applications to sputum and bronchoalveolar lavage fluid highlight its potential in diagnosing respiratory infections, particularly in critically ill patients [71]. Even easily accessible, non-invasive samples such as saliva have been explored in preliminary studies, opening perspectives for rapid and less invasive diagnostic approaches [72].

Despite these encouraging results, several critical issues remain, particularly the lack of protocol standardization. Studies reviewed here reveal variability in excitation wavelengths, SERS substrates, sample preparation methods, and analytical algorithms, limiting inter-study comparability. Moreover, spectral overlap among phylogenetically related species may result in classification mistakes, especially in the absence of wide, well-balanced datasets.

Additional considerations concern the biological interpretability of spectral signatures. In some cases, discrimination between resistant and susceptible strains appears linked to biochemical differences not directly associated with the molecular mechanisms of resistance [55]. While this probably does not diminish the diagnostic utility of the technique, it warrants caution in pathophysiological interpretation of the findings. Therefore, transitioning from proof-of-concept to a clinically deployable tool requires harmonization of key technical parameters. The standardization of excitation wavelengths—either 532 nm (high energy, optimal for biomolecules) or 785 nm (lower fluorescence, preferable for clinical samples)—is essential to ensure database compatibility. The development of certified SERS substrates with minimal coefficients of variation is likewise necessary.

Finally, a major step toward clinical translation involves validating these models on external, multicenter cohorts with diverse genetic and ethnic backgrounds, thereby moving beyond controlled preclinical research settings.

## 6. Conclusions

Raman spectroscopy is emerging as a technological platform with significant potential for sepsis management. It offers the capability to identify the pathogen and monitor host metabolic distress within a single analytical framework that is, theoretically, rapid and culture-independent.

Despite this cautious optimism, the gap between controlled laboratory settings and the complex environment of real-world emergency department scenarios cannot be overlooked. At present, any claim that this technology could radically transform empirical antibiotic strategies is premature. Much of the available evidence remains limited to single-center preclinical studies with relatively small sample sizes.

The future of Raman spectroscopy in sepsis lies in an integrated multimodal approach, in which spectroscopy provides rapid biochemical signatures while artificial intelligence ensures robust and in-depth interpretation.

Large-scale validation in real-world clinical settings therefore represents an essential prerequisite before routine implementation can be considered. Translation from bench to bedside will require well-designed prospective multicenter trials, methodological harmonization, and rigorous evaluation of cost-effectiveness.

Only through such a structured pathway will it be possible to clearly define the role of Raman spectroscopy in the future of sepsis diagnostics.

This review aims to raise awareness among experts in the field and to encourage the application of this emerging technology in clinical studies to assess its potential in accelerating sepsis diagnosis.

A key question remains whether, if successfully validated, Raman spectroscopy may represent a fundamental step forward toward rapid, precise, and individualized diagnosis, with potential benefits extending to improved patient outcomes, more appropriate therapeutic management, reduced length of hospital stay, and overall containment of healthcare costs.

## Figures and Tables

**Table 1:** Studies employed in this review.

Total Studies Identified for the Preparation of the Review			72
Results section	In vitro studies		23
	Preclinical studies	Animal models	3
	Prospective studies	Human subjects	2

**Table 2:** Immunology in Raman spectroscopy: in vitro study in the field of nanobiotechnology and biomedical diagnostics.

Authors	Type	Year	Samples	Results	Wavelength
Ying Wang et al. [32]	In vitro study in the field of nanobiotechnology and biomedical diagnostics	2023	/	In this study, surface-enhanced Raman scattering (SERS) was used for the joint detection of sepsis biomarkers interleukin 6 (IL-6) and procalcitonin (PCT).	785 nm
Anh H Nguyen et al. [33]	In vitro study in the field of nanobiotechnology and biomedical diagnostics	2016	/	SERS technology was used for triplex assay analysis (PCR, PCT, sTREM-1) in the diagnosis of sepsis	785 nm
I Olaetxea et al. [36]	In vitro study in the field of nanobiotechnology and biomedical diagnostics	2019	12 blood samples and 12 samples of PBS	Raman spectroscopy enabled the quantification of lactate and pH values in blood and in solutions, with a mean prediction error of 0.3 mM for lactate and 0.08 units for pH, respectively.	785 nm
Xiaomei Wang et al. [34]	In vitro study in the field of nanobiotechnology and biomedical diagnostics	2021	/	SERS-based magnetic immunoassay technique for the detection of IL-6 and PCT	785 nm
Ying Wang et al. [35]	In vitro study in the field of nanobiotechnology and biomedical diagnostics	2023	/	This study combines the SERS technique and magnetic materials for the detection of IL-6	785 nm

**Table 3:** Immunology in Raman Spectroscopy: Preclinical studies conducted in animal models.

Authors	Type	Year	Samples	Results	Wavelength
**Osadare et al.** [37]	Preclinical study conducted in an animal model	2023	36 mice	In a septic context, splenocytes show important spectral variations in DNA, with minor abnormalities also found at the protein and lipid levels.	532 nm
**Meiyan Wu et al.** [38]	Preclinical in vivo study in an animal model	2020	1° experimental phase 7–29 mice; 2° experimental phase 45 mice; 3° experimental phase 5–8 mice in each of the two groups (group with a value greater than 1.059 and group with a value less than 1.059	In vivo study of mitochondrial redox status by RRS allows for a rapid diagnosis of sepsis and shows greater prognostic accuracy with respect to changes in blood lactate levels.	532 nm

**Table 4:** Immunology in Raman spectroscopy: prospective study conducted in human models.

Authors	Type	Year	Samples	Results	Wavelength
**Ramoji A et al.** [39]	Prospective, nonrandomized, monocenter observational, human study	2021	24 patients with sterile inflammation, 19 infected patients, 18 patients with sepsis	Raman spectroscopy can study the leukocyte activation state in hospitalized patients with inflammation, infection and sepsis.	785 nm

**Table 5:** Raman spectroscopy for the assessment of pathogen susceptibility to antimicrobial agents.

Author	Type	Year	Samples	Results	Wavelength	CFU
Kang et al. [46]	In vitro diagnostic validation study	2024	130 blood culture bottles	*E. coli* with 43 isolates *K. pneumoniae* with 28 isolates *A. baumannii* with 16 isolates *E. faecium* with 10 isolates *E. faecalis* with 7 isolates *S. aureus* with 13 isolates *P. aeruginosa* with 13 isolates	785 nm	10^9^ CFU/mL
Dekter et al. [40]	In vitro diagnostic validation study	2017	133 bacterial isolates from blood culture	Comparable sensitivity to VITEK 2.	785 nm	500 CFU/8mL
Han et al. [41]	In vitro diagnostic validation study	2023	164 bacterial isolates from blood culture	SERS accurate in assessing antibiotic response	632.8 nm	3 × 10^9^ CFU/mL
Schroder et al. [42]	In vitro diagnostic validation study	2015	15 bacterial isolates from blood culture	Performing Raman spectroscopy in the detection of vancomycin resistance	532 nm	10^8^ CFU /mL
Yi et al. [44]	In vitro diagnostic validation study	2021	9 urine and 3 blood samples	Antibiotic resistance findings in sepsis and urinary tract infection	532 nm	5 × 10^5^ CFU/mL
Assman et al. [43]	In vitro diagnostic validation study	2015	/	Vancomycin resistance observed after 90 min	532 nm	10^8^ CFU /mL

**Table 6:** Raman spectroscopy for pathogen identification.

Author	Type	Year	Samples	Results	Wavelength	CFU
Rebrosova et al. [52]	In vitro diagnostic validation study	2023	305 microbial strains belonging to 28 species from blood culture	Raman tweezers could allow the pathogen to be detected directly in the blood as a point of care.	785 nm	/
Nakar et al. [50]	In vitro diagnostic validation study	2022	25 bacterial isolates from blood culture	UVRR spectroscopy is more effective than SC-RMS in classifying *E. coli* and *Klebsiella* isolates at the genus level. Furthermore, it can probably also distinguish between infections caused by *K. oxytoca* and *K. Pneumoniae*.	244 nm	10^8^ CFU/mL
Pistiki et al. [55]	In vitro experimental proof-of-concept study	2022	Clinical isolates of MRSA and MSSA	For the discrimination of MRSA and MSSA isolates, the best technique would appear to be single-cell analysis with excitation at 532 nm.	532 nm or 785 nm	10^8^ CFU/mL
Dhams et al. [56]	Comparative experimental study	2022	59 isolates of patients with Streptococcus	Discriminatory ability of pneumococcus from another streptococcus by only 70%	632.8 nm	/
Pilat et al. [53]	In vitro experimental proof-of-concept study	2018	Isolate di *E. coli* 683 from blood culture	Identification using Raman tweezers.	532 nm or 785 nm	10^6^ cells/mL
Li et al. [47]	Preclinical in vivo study in an animal model	2020	40 rats	SERS-based combined with PCA-LDA has good diagnostic performance in *T. spiralis infection*.	785 nm	3500 muscle larvae (ML)
Hassan et al. [48]	Ex vivo case–control study on human blood samples.	2025	723 clinic samples	98.84% pathogen identification.	785 nm	3 μL of blood
Kaushik et al. [49]	In vitro study in the field of nanobiotechnology and biomedical diagnostics	2025	/	SERS enables the detection of bacteria at a concentration of $10^{2}$ cfu/mL.	532 nm	a maximum of 10^8^ CFU/mL, down to a minimum of 10^2^ CFU/mL
Park et al. [54]	Experimental proof-of-concept pilot study	2025	*E. coli* samples, both in culture and in aqueous suspension	*E. coli* detection with SERS and Acustofluidics integration	633 nm	1.75 × 10^5^ CFU/mL
Alagar et al. [58]	In vitro experimental proof-of-concept study	2025	7.5 mL of contaminated whole blood in vitro	Quantification of seven Candida species in 7.5 mL of whole blood by SERS	785 nm	2 CFU/mL
Effah et al. [57]	In vitro diagnostic validation study	2023	Food and clinical samples	The SPION-PEI-Au-Van nanocomposite has a capture efficiency of 78.1% for KP and 75.2% for AB.	785 nm	10 cells/mL
Aubrechtová Dragounová et al. [60]	Ex vivo diagnostic experimental study	2023	59 urine samples	50% of samples with mixed infections: in 18 samples, two bacteria, in 11 samples, three or more bacteria.	532 nm	Bacterial load varies based on the actual sample
Pezzotti et al. [59]	In vitro Diagnostic Validation Study	2022	/	species-level identification of Candida from cultured colonies	532 nm	/
De plano et al. [51]	In vitro Diagnostic Validation Study	2019	/	Magnetic separation using M13 phage-coated beads has a detection limit of 10 Colony-forming Units per 7 mL of blood.	785 nm	10 CFU/7 mL

**Table 7:** Comparison of techniques for pathogen identification in blood.

	MALDI-TOF	PCR	Raman Spectroscopy
**Principle**	Analysis of protein profiles using mass spectrometry	Amplification of DNA/RNA	Utilizes the Raman effect for molecular characterization
**Response Time**	1–2 h on positive blood cultures	3–6 h directly from the sample	From 20 min to 2 h
**Sensitivity and Specificity**	High for bacteria; lower for fungi and polymicrobial infections	Very high but species-specific	High, including for non-target pathogens
**Applicability**	Positive cultures	Biological samples	Biological samples
**Additional Information**	Enables resistance detection	Simultaneous detection of multiple microorganisms in the same sample	Enables resistance detection, evaluates metabolic status, and is applicable to immune cells

**Table 8:** Advantages and Disadvantages of Raman Spectroscopy.

Raman Spectroscopy	
Advantages	Disadvantages
**Rapid Turnaround Time (TAT)**	Standardization Gaps
**Low Sample Volume**	Reproducibility Issues
**Antibiotic Independence**	Database Dependency
**Label-free & non-destructive**	Phylogenetic Overlap
**Versatility**	Preclinical Status
**Direct Analysis**	Operational Complexity

## Data Availability

No new data were created or analyzed in this study. Data sharing is not applicable to this article.
